# Systematic Review of Nomograms Used for Predicting Pathological Complete Response in Early Breast Cancer

**DOI:** 10.3390/curroncol30100662

**Published:** 2023-10-16

**Authors:** Marcelo Antonini, Gabriel Duque Pannain, André Mattar, Odair Ferraro, Reginaldo Guedes Coelho Lopes, Juliana Monte Real, Lucas Miyake Okumura

**Affiliations:** 1 Mastology Department, Hospital do Servidor Público Estadual, Francisco Morato de Oliveira, São Paulo 04029-000, Brazil; gabrielduquep@gmail.com (G.D.P.); odairferraro@iamspe.gov.br (O.F.); jarelu@uol.com.br (R.G.C.L.); juliana_mreal@yahoo.com.br (J.M.R.); 2 Mastology Department, Women’s Health Hospital, São Paulo 01206-001, Brazil; mattar.andre@gmail.com; 3 Statistiscs Department, Value ArchTech, Curitiba 80250-080, Brazil; okumura.lucas@gmail.com

**Keywords:** breast neoplasms, cancer, nomograms, neoadjuvant therapy, systematic review

## Abstract

Pathological complete response (pCR) is an important surrogate outcome to assess the effects of neoadjuvant chemotherapy (NAC). Nomograms to predict pCR have been developed with local data to better select patients who are likely to benefit from NAC; however, they were never critically reviewed regarding their internal and external validity. The purpose of this systematic review was to critically appraise nomograms published in the last 20 years (2010–2022). Articles about nomograms were searched in databases, such as PubMed/MEDLINE, Embase and Cochrane. A total of 1120 hits were found, and seven studies were included for analyses. No meta-analysis could be performed due to heterogeneous reports on outcomes, including the definition of pCR and subtypes. Most nomograms were developed in Asian centers, and nonrandomized retrospective cohorts were the most common sources of data. The most common subtype included in the studies was triple negative (50%). There were articles that included HER2+ (>80%). In one study, scholars performed additional validation of the nomogram using DFS and OS as outcomes; however, there was a lack of clarity on how such endpoints were measured. Nomograms to predict pCR cannot be extrapolated to other settings due to local preferences/availability of NAC. The main gaps identified in this review are also opportunities for future nomogram research and development.

## 1. Introduction

Breast cancer (BC) is one of the most common diseases in women [1]. In 2020, 2.3 million BC cases were diagnosed, and it is believed that nearly 65% of cases were considered early breast cancer [2]. Neoadjuvant chemotherapy (NAC) has been established as a relevant treatment modality for high-risk localized disease and an unresectable or breast-conserving approach; in tandem, pathological complete response (pCR) has emerged as a relevant surrogate outcome to assess the effects of NAC [3,4].

One recently published systematic review with meta-analyses included definitions regarding the association of pCR and definitive outcomes, such as overall survival and disease-free survival. The meta-analyses [5] revealed that pCR after NAC was associated with increased overall survival (HR = 0.22; 95% PI, 0.15–0.30) and event-free survival (HR = 0.31; 95% PI, 0.24–0.39), specifically for triple-negative (HR = 0.18; 95% PI, 0.10–0.31) and HER2+ (HR = 0.32; 95% PI, 0.21–0.47) diseases.

Considering the importance of pCR during NAC and its impact on overall survival, mathematical models between baseline patient characteristics and the outcome of interest were developed (nomograms) [6,7] to predict ways in which clinical information (prior data to NAC) would be able to predict pCR. The objective of the nomograms is to maximize pCR likelihood [8], ensuring that a given patient is only exposed to NAC if they have the highest chances of benefitting from systemic chemotherapy.

Although there is evidence of NAC importance and its relation with pCR [5], it is not known how these nomograms were developed, nor is the external validity of such tools well appraised in the literature. That is, there is a gap in establishing whether such nomograms were created considering good practices for modeling predictive equations. Considering this, the purpose of present systematic review is to describe the current nomograms that predict pCR, explore whether they comply with good practices of modeling research, and assess the quality, source and validity of the predictive tools for clinical practice.

## 2. Methods

### 2.1. Protocol Registration and Rationale of Review

Our review followed the PRISMA statement, and its protocol was registered at PROSPERO/University of York, which can be accessed online (https://www.crd.york.ac.uk/prospero/ (accessed on 14 May 2022), with protocol number: CRD42022330037).

The strategy for manuscript finding included the use of indexed keywords (“breast neoplasm” AND “nomogram”) with no language restrictions. We only included studies published from January 2010 to June 2022 as earlier evidence might be outdated regarding chemotherapies available for neoadjuvant schemes, including anti-HER2 targeted antibodies.

There were five research questions for this review:

1. What nomograms are available in the literature for predicting pathological complete response (PCR) in early-stage breast cancer?
2. What are the clinical characteristics from the studied population?
3. How were such nomograms validated?
4. Did nomograms use an established database (administrative data of procedures) or were they validated with a cohort built for the purpose of developing a nomogram with clear inclusion and exclusion criteria?
5. Are the nomograms still valid for clinical use? If so, what are the current gaps to be addressed in future studies?

### 2.2. Data Sources and Searches

In total, four databases were searched: PubMed/MEDLINE, Embase, Cochrane Central Register of Controlled Trials, and Lilacs. Gray literature was also assessed through Google Scholar. For definition purposes, pathological complete response (PCR) was defined as both absence of invasive cancer in the breast and no residual disease in the axillar region [9].

### 2.3. Study Selection and Data Extraction

The main eligibility criteria from the review included studies about nomogram creation in early female breast cancer. We excluded studies that did not assess PCR, abstracts with no complete text published and those with PCR restricted to residual cancer only in the breast.

Screening (selection by title and abstract reading) was performed by two independent reviewers who performed eligibility (inclusion by full text reading) and data extraction. In case of discrepancies between the two, a third reviewer was invited to decide whether the study should or should not be included.

The following data were extracted: (a) general study information (country in which the research was developed, data source for nomogram development, and study inclusion and exclusion criteria); (b) BC characteristics (age, stage, neoadjuvant schemes, histologic grade, TNM, hormonal receptors and HER2 status); and (c) the nomogram creation process (definition of predictors and pCR, use of additional outcomes such as overall survival and disease-free survival, follow-up period, missing data handling and statistical methods for nomogram development and validation).

All aforementioned data extraction was planned based on a tool used for assessing clinical prediction models [10].

### 2.4. Data Synthesis and Analysis

A descriptive analysis was performed to assess each of the included manuscripts by summarizing them in tables. Data were synthesized and analytically displayed specifically to answer the five questions stated for the review. No meta-analyses were performed because there were incomplete data for recalculating the diagnostic odds ratio, hierarchical summary receiver operating characteristic (HSROC) and other statistical estimates.

## 3. Results

**Figure 1:**
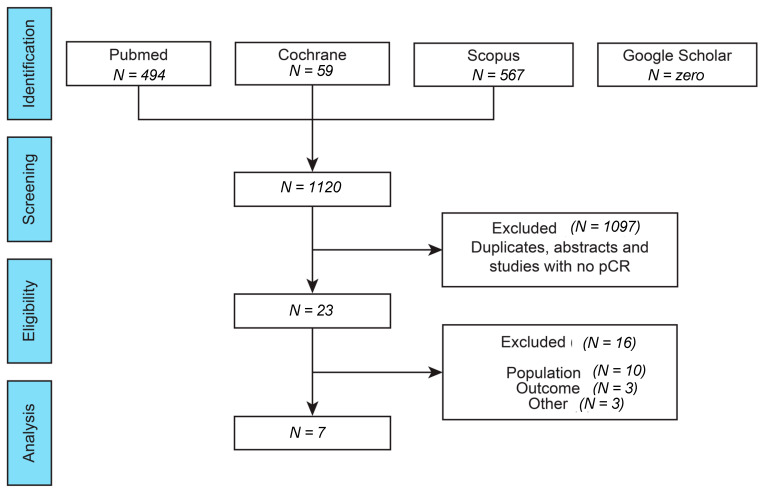
Study selection flowchart.

A total of 1120 studies were identified. Nevertheless, 1097 titles were excluded due to not being compatible with our preestablished inclusion criteria. During the eligibility phase (full text reading), only seven studies [8,11,12,13,14,15,16] were included for complete text reading and analyses (Figure 1). Overall, this review included 3822 women with early-stage BC.

### 3.1. Overall and BC Characteristics from Studies

Considering the seven manuscripts included in the analyses, only one study was performed in the USA, while the other six were performed in the Republic of Korea or China. All studies were nonrandomized retrospective cohorts. The inclusion and exclusion criteria revealed that none of the studies included solely early-disease BC (Stage 1 or 2). That being said, most of the studies had patients with Stage 1 and 2 disease as the predominant population (Table 1).

Most BC patients were between 40 and 50 years old, with heterogeneous patterns of tumor types. Despite our efforts to focus on one breast cancer subtype specifically, only one study was composed of an exclusive population with the same tumor type (triple-negative), and all other studies had heterogeneous populations with different tumor types. The neoadjuvant chemotherapies prescribed were also diverse, with one study presenting markedly low rates of taxane use (less than 26% received a taxane-derived therapy). In contrast, Kim et al. [11] and Li et al. [8] presented a 97 to 100% use of taxanes. Markedly, two studies had significantly low rates of anti-HER2 use due to cohort inclusion. The Kim et al. [11] development nomogram cohort had a 37% anti-HER2 use, while the Zhang et al. [15] sample was composed only of patients with TNBC. On the other hand, 75% of the Fujii et al. [14] sample consisted of patients using double anti-HER2 blockage therapy (pertuzumab + trastuzumab) (Table 2).

When reported, studies included 46 to 48% of high-grade histologic BC, and more than 90% of the samples were ductal carcinomas. Since Ki-67 is a marker used in the immunohistochemical evaluation of BC, it was also used to characterize the evaluated tumors. It is a substance released during cell division. Therefore, tumors that divide more frequently have higher Ki-67. Of note, Ki-67 cutoffs varied substantially between studies. Hwang et al. [12] and Hou et al. [13] demonstrated the same thresholds for Ki-67 (> or <20), and 30% of patients had Ki-67 < 20; another study from Li et al. [8] reported a Ki-67 threshold of 65, suggesting that 76% of the included population was Ki-67 < 65 (Table 3).

### 3.2. Nomogram Development and Assessment of How They Were Developed

The majority of the studies (six out of seven) included adequate reporting of the predictors that would be included in the regression analyses, but only two included the definition of PCR used as an outcome. Overall pCR varied from 13 to 56% of the sample studies, while the non-pCR population varied from 44 to 85% of the included population. Only one study included additional outcomes to assess the validity of the nomogram against hard outcomes, such as disease-free survival and overall survival, while the methods used to measure these endpoints were not detailed, there were gaps in defining censoring, follow-up procedures, and the confirmation of outcomes, especially since most of these studies were retrospective) [12] (Table 4).

**Table 1:** Overall characteristics of the included studies.

Author–Year	Country	Inclusion Criteria	Exclusion Criteria	Other Relevant Information
Kim et al., 2021 [11]	Republic of Korea	Stage 2 and 3 BC submitted to neoadjuvant chemotherapy, consecutive patients from January 2011 to December 2017	Lack of imaging (magnetic resonance) or mammography (before or after neoadjuvant scheme); bilateral cancer; previous history of cancer.	Use of anti-HER2 therapies increased from 2014
Hwang et al., 2019 [12]	Republic of Korea	Not described. It is suggested that patients with BC treated with NAC and followed by surgical resection and that had histologic evaluation were included	Not described.	A significant part of the cohort (248) had BC treated with NAC and followed by surgery between 2004 to 2013. Additional 60 patients treated between 2016 and 2017 were included because they received double anti-HER2 therapy
Li et al.,2021 [8]	China	Unilateral primary invasive breast cancer diagnosed by biopsy in patients with age between 18 and 70 years, clinical stage: 2 or 3, which met the requirements of the 2019 NCCN guidelines for NAC of breast cancer	Patients in the process of treatment, incomplete pathological results, receiving nonstandard neoadjuvant chemotherapy or surgery.	Does not apply
Hou et al., 2020 [13]	China	Not clearly described	1. Bilateral BCs or meta-synchronous primary malignancies; Stage 0 and Stage 4 BC. 2. Patients already treated outside our hospital; unavailability of variables we wanted to include.	Does not apply
Fujii et al., 2017 [14]	USA	Partially described Stages 1–3 HER2-positive invasive breast cancer patients who had definitive surgery in 1999–2015 and received NST	Patients for whom continuous ER and PR levels or HER2/CEP17 ratios were not available were excluded from analysis.	Does not apply
Zhang et al., 2019 [15]	China	Female, histologically and molecularly confirmed to have TNBC before NAC, and received four cycles of anthracycline (epirubicin or adriamycin) and cyclophosphamide followed by four cycles of taxane every 3 weeks before surgery	Previous or concurrent cancer, bilateral breast cancer, or distant metastases.	Does not apply
Jin et al., 2016 [16]	China	Patients diagnosed with primary breast cancer and who received neoadjuvant chemotherapy followed by standard surgery were enrolled	Patients with HER2-positive core needle biopsy samples, with metastatic disease, with missing data or with previous endocrine therapy were not eligible for this study. Patients missing relevant information, who were HER2-positive or who had received neoadjuvant chemotherapy regimens other than cyclophosphamide, epirubicin and 5-fluorouracil, cyclophosphamide, epirubicin and 5-fluorouracil followed by paclitaxel or docetaxel and epirubicin, navelbine and epirubicin or paclitaxel and carboplatin or paclitaxel and cisplatin were excluded from our study.	Does not apply

Notes: BC—breast cancer, NAC—neoadjuvante chemotherapy. Number of centers involved in study is 1. Retrospective cohort-based, nonrandomized sample is used for data source for developing the nomogram.

**Table 2:** Clinical information from studies.

Author–Year	Demographics	Clinical Staging	Neoadjuvant Schemes	Histologic Grade/Type	Receptor Status	Other Relevant Information
Kim et al., 2021 [11]	49 ± 10 years old	T1/T2 60% N0-N1 45%	97% taxane-based	High grade: 46%	HER2: 34%	Anti-HER2 therapy was higher in developing cohort (37%) than validation cohort (94%). Ki-67: 15 (1–90)
Hwang et al., 2019 [12]	~73% were <50 years old	~38% cT1-2 ~62% cT3-4 ~21% cN0-1 ~79% cN2-3	60% taxane-based 40% anthracycline-based 60% > 4 cycles of NAC	~48% Grade 1–2 ~52% Grade 3 91% Ductal type ~9% others	~19% Lu A ~28% HER2− Lu B ~10% HER2+ Lu B ~14% HER2+ ~30% TN	The 60 patients who received double anti-HER2 block, but that population was not described anywhere except from methods. Ki-67 < 20 was 30%
Li et al., 2021 [8]	47 ± 10 years old	2 77% and 3 23% 87% cT0-2 and 13% cT2 > 2 79% N0-1 and 21% N2-3	100% contained taxanes 53% contained anthracycline 46% contained anti-HER2 3% contained double anti-HER2 block 83% > 5 NAC cycles	Not included	32% HER2− Lu B 7% HER2+ Lu B 44% HER2+ 17% TN	Ki-67 < 65 was 76%
Hou et al., 2020 [13]	More than 40% were >49 years old	90% T1-2	26% taxane and anthracycline-based schemes	Not included	76% HER2-negative	Ki-67 > 20 was 69%
Fujii et al., 2017 [14]	49 years old (range 19–84)	2 50% and 3 47%	Cytotoxic agents alone 15% Anti-HER2 based 10% Double anti-HER2 75%	Ductal 94% Lobular 2%	Unclear, but it is suggestive that based on anti-HER2 therapies, the sample was largely composed of HER2+	Does not apply
Zhang et al., 2019 [15]	49.5 (33.1–64.0, IC95%)	cT1-2 80% cN0-1 62.5%	Eight cycles of thrice-weekly standard NAC (anthracycline and cyclophosphamide followed by taxane)	Not described	The sample was composed of TN	The sample was composed of TN
Jin et al., 2016 [16]	80% were >40 years old	T1-2: 53% T3-4: 47% N0-1: 94% N2-3: 6%	Median of 4 cycles (range, 1–6 cycles): navelbine and epirubicin, cyclophosphamide, epirubicin and 5-fluorouracil, paclitaxel with carboplatin/paclitaxel with cisplatin or epirubicin and 5-fluorouracil followed by paclitaxel or docetaxel and epirubicin	Not described	Not described	Does not apply

**Table 3:** Dependent and independent variables used for the nomogram.

Author–Year	Predictors from Nomogram	Outcome from Nomogram	Other Relevant Outcomes	Other Relevant Information
Kim et al., 2021 [11]	Not described	pCR. Manner of assessment not described in detail	Not applicable	Not applicable
Hwang et al., 2019 [12]	Pre-NAC TIL level, age, menopausal status, tumor size, clinical nodal stage (cN), histologic grade, NAC regimen and cycle number, expression level of ER, PR, HER2, and Ki-67	pCR 15% and non-PCR 85%	Disease free survival and breast cancer-specific survival, as means to assess the prognostic value of post-NAC TILSs. Five-year BCSS rate was 45.6%, and 5-year DFS rate was 38.3%	High post-NAC TILs and low Ki-67 index were significant predictors of BCSS and DFS in the multivariable model. DFS and BCSS had undetailed definitions about censoring, follow-up and criteria for “disease-free” or “breast cancer-related mortality”
Li et al., 2021 [8]	Body mass index, Carbohydrate antigen 125, Total protein, Blood urea nitrogen, Cystatin C, Potassium, Phosphorus, platelet distribution width, activated partial thromboplastin time, thrombin time, antibody of hepatitis B surface	pCR 35.4% and non-pCR 64.6%	Not applicable	Not applicable
Hou et al., 2020 [13]	Menopause status, family history of BC, initial tumor size, estrogen receptor status, HER2/neu status, and Ki67 expression	Training set pCR 30% Validation set pCR 23%	Not applicable	Not applicable
Fujii et al., 2017 [14]	Variables of interest included age, race, BMI prior to NST, menopausal status, histologic findings, clinical stage, IBC or nonIBC, ER expression, PR expression, HER2/CEP17 ratio, and NST regimen (containing TmAb, TmAb plus pertuzumab (PmAb), or cytotoxic agents only)	pCR 56% and non-pCR 44% pCR after NST was defined as no evidence of residual invasive cancer in the breast and no residual cancer in the axilla at the time of definitive surgery	Not applicable	Not applicable
Zhang et al., 2019 [15]	Clinical tumor stage, lymphocyte to monocyte radio, fibrinogen level, D-dimer level	pCR 48.8% and non-pCR 51.2%	Not applicable	The sample was composed of TN
Jin et al., 2016 [16]	Tumor size, hormone receptor status, neoadjuvant chemotherapy regimens, cycles used, age, menopausal status, nodal status	pCR was defined as complete disappearance of invasive carcinoma in the breast and regional lymph nodes pCR 13% and non-pCR 77%	Not applicable	Not applicable

**Table 4:** Methodologic assessment of the nomogram development process.

Author–Year	Cohort for Development and Validation	Sample *(n*, Adequate Report of Participants, Covariates and Outcomes)	Missing Data	Statistical Methods	Final Prediction Model Specified, Including 95% CI?
Kim et al., 2021 [11]	Yes	Development (*n* = 359) and validation (*n* = 351) There was not sample report for covariates and outcomes	Not described; however, according to baseline characteristics, there were no missing data	Uni- and multivariate analyses based on logistic regression. Predictors included based on *p* < 0.1. Calibration based on slope (1 = perfect, <1 overfitted). Interobserver independent validation (kappa and interclass correlation).	Yes. AUC 0.9 (IC95% de 0.86 a 0.94)
Hwang et al., 2019 [12]	Not described	The sample was 248 pair-matched pre-NAC biopsy and post-NAC resection	Not described; however, according to baseline characteristics, there were no missing data	Univariable logistic regression model and backward stepwise selection for final multivariable model were conducted. Calibration was assessed graphically.	Unreported, although it was described in methods
Li et al., 2021 [8]	Unclear	The sample consisted of 130 patients. All covariates and predictors had their respective number of patients	Not described; however, according to baseline characteristics, there were no missing data	Univariable analysis and multivariable binary logistic regression were used to determine independent predictors of bpCR after NAC. The nomogram was developed using a multivariable logistic regression model. Calibration of the nomogram was carried out by the 1000 bootstrap resampling internal verification and was displayed by the calibration curve. GiViTI calibration band: agreement between predicted and observed probability. Brier score: prediction accuracy.	Undescribed. AUC was 0.941 (95% confidence interval: 0.900–0.982)
Hou et al., 2020 [13]	Yes	Development (*n* = 689) and validation (*n* = 357). All covariates and predictors had their respective number of patients	Tumor grade was missing, and that is why it was not included as covariate	Univariate logistic regression analysis was conducted on variables in the training set, and variables with *p* < 0.05 were included in multivariate logistic regression. External validation was performed on the nomogram. The unbiased prediction of pCR by the nomogram was ensured by drawing the calibration curve.	Undescribed; AUC was 0.753 (95% confidence interval: 0.718–0.788)
Fujii et al., 2017 [14]	No	The sample was 793	Not described; however, according to baseline characteristics, there were no missing data	Associations between categorical variables were examined using the χ^2^ test or Fisher exact test when appropriate. The Wilcoxon rank-sum test or Kruskal–Wallis test was used to examine differences in continuous variables between or among patient groups. Multivariate logistic regression models were applied to assess the effect of variables of interest on pCR status. Backward stepwise selection was applied to determine which variables were included in the final multivariable model. A nomogram was built to estimate the probability of pCR given the risk factors in the final multivariable model. A bootstrap validation method based on 200 bootstrap samples was employed to estimate the bias-corrected or overfitting-corrected predictive discriminative ability of the model, which was presented as the concordance index.	Not reported
Zhang et al., 2019 [15]	No	The sample was 80	Not described; however, according to baseline characteristics, there were no missing data	The optimal cut-off values of the laboratory indexes were determined by the Youden index using receiver operating characteristic (ROC) curve analyses. Forward stepwise logistic regression (likelihood ratio) analysis was applied to identify predictive factors for a pCR of NAC. A nomogram was then developed according to the logistic model, and internally validated using the bootstrap resampling method.	AUC was 0.803 (95% confidence interval 0.706–0.899
Jin et al., 2016 [16]	Yes	The 815 were randomized to a training (500) and validation set (315)	Missing data on chemotherapy was an exclusion criterion.	Chi-square test was used to evaluate the relationship between neoadjuvant chemotherapy regimens and other characteristics. Fisher’s exact test was performed when necessary. All reported *p*-values are two-sided.	AUC from validation set: 0.703 (95% CI: 0.624–0.782)

Regarding the methodological assessment of the nomograms developed, no studies included reports on the number of days patients were retrospectively followed. Only three studies included cohorts for nomogram development and validation. Few studies included information on ways the missing data were handled, although most of them indirectly indicated that inclusion criteria depended on complete clinical information available in the retrospective chart review. Finally, only one study included reporting of the prediction model (equation), and six studies provided the area under the curve (AUC) from the final nomogram, which varied from 0.706 [16] to 0.941 [8] (Table 4, Supplementary File S1).

## 4. Discussion

To our knowledge, this is the first systematic review of studies about nomograms developed to predict pathological complete response in women with breast cancer. Overall, we identified that studies are not generalizable to other settings and that their validity might be affected by diverse methodological flaws. We highlight the following: low pCR prediction variables entering the studies, low clinical applicability for 2022 and questionable quality of the validation process of nomograms.

### 4.1. Low pCR Prediction Variables in the Nomogram

Many studies included covariates that offered no prediction of pCR in the final nomogram [8,16]. For example, baseline characteristics, such as age (pCR vs. non-pCR age, 46.2 ± 9.9 vs. 48.3 ± 10.5, *p* value = 0.234) [8] and tumor size (using T1 as a reference, T2 (*p* value = 0.754), T3 (*p* value = 0.104) and T4 (*p* value = 0.577)), were used as components of the final equation. Although many variables, such as tumor size (T1–T4), can be logically related to predicting pathological response, the study did not demonstrate such an association.

The concern of adding covariates that have low predictability of pCR is also worsened by the numerical manipulation of continuous variables to categoric variables, such as Ki-67 levels. In our systematic review, we found that Ki-67 levels were frequently correlated with pCR [8,13,16], but cutoff levels were often different between studies (65% and 20%) [8,13].

A recent meta-analysis published in 2017 showed that cutoff values to define “high” and “low” Ki-67 levels varied between 10 and 50% [17]. Additionally, when it was expected that higher levels of Ki-67 would increase the chance of predicting pCR, in the same publication, there was no evidence of an increasing chance of higher pCR by analyzing subgroups of different cutoffs (metanalyses did not show a “dose–response” relation between categories of Ki-67 levels (≤14%, 15 to 29% and ≥30%) and pCR (OR = 5, 3.7 and 3.5, respectively) [17].

Such frailty in defining ideal Ki-67 levels as high or low levels might markedly impact final decision making, especially when building a nomogram. Although this review does not aim to suggest the optimal cutoff, we identified relevant sources of inconsistencies that should be well explored in further nomogram development research [18].

### 4.2. Clinical Applicability for 2022

Interestingly, most of the nomograms that included the HER2+ population had low exposure to anti-HER2 therapies, specifically dual HER2 blockade [11,12] or even single-anti-HER2 therapy [11]. In the NeoSphere trial [19], the benefits of adding pertuzumab to trastuzumab were shown 4 years before the publication of the nomograms [11]. Any review of predictive models, such as nomograms, needs to take into account the updates in systemic therapy, especially in HER2-positive and triple-negative tumors. With the rapid change in systemic therapy, previous nomograms may become outdated fairly quickly. This explains why none of the studies mention any emerging biomarkers in breast cancer or even established markers such as ER/PR and HER2.

In addition, six out of seven studies were conducted using cohorts of Asian BC patients. On the one hand, nomograms provide high validity for Asian populations who are diagnosed at ages between 40 and 50 years, and the mortality risk is higher; on the other hand, it is known that Western countries have the opposite characteristics: women are diagnosed at 60 to 70 years of age, and the mortality is decreasing [20].

### 4.3. Quality of the Validation Process

Finally, the nomograms are poorly compliant with all steps of the validation process. It was noted that Jin et al. [16], Hou et al. [13] and Kim et al. [11] used development and validation samples to develop the pCR predictive equation. Some of the studies did not include clearly reported outcomes for pCR and non-pCR or include descriptive details about all included covariates and their relation with pCR; in addition, the definition of pCR used and how it was assessed based on retrospective data collection was not confirmed in many cases. In addition to these methodological concerns, many studies also did not assess the relation between pCR and other outcomes, such as invasive disease-free survival or even overall survival.

The TRIPOD checklist is a statement that contains a minimal set of information that should be reported in prediction (nomogram) studies [10]. As the data extraction of the present review was inspired by this checklist, the development and validation process involved considering the TRIPOD statement point of view.

## 5. Limitations

This review is not absent of limitations. The quality of the nomogram studies was considered relative to TRIPOD, which is a checklist created to help assess studies on prediction tools. It was neither specifically designed to assess cancer studies nor to evaluate nomograms for early BC. However, more than having methodological adequation, clinical validity should be the key driver for assessing the applicability of the nomograms. In this sense, the studies included in this review might be useful for settings that use the same therapies or might be compatible with the studied population of the nomograms.

## 6. Conclusions

Nomograms to predict pathological complete response might only be valid for extrapolations to other settings if there is clear understanding that most of them were developed for Asian populations and reflect locally available therapies, which might be overpromoted depending on the year of publication. The main gaps identified in this review are also opportunities for future nomogram research. There is a need for better definitions related to the ways in which covariates can be manipulated to be included in the nomogram, how local health care system can affect the external validity of the nomograms, and how quality of the data included is able or not able to predict pCR as surrogate outcome, final outcome, disease-free survival and overall survival.

## Supplementary Materials

The following supporting information can be downloaded at: https://www.mdpi.com/article/10.3390/curroncol30100662/s1, File S1: Annexx—Nomograms reported in the studies [8,11,12,13,14,15,16].
