# Supplementary material for: Systematic Review of Nomograms Used for Predicting Pathological Complete Response in Early Breast Cancer
Source: Curr Oncol. 2023 Oct 16;30(10):9168–80. doi: 10.3390/curroncol30100662 (PMC10605609; doi:10.3390/curroncol30100662)

File S1 Annexx – Nomograms reported in the studies.

[11]Kim et al. Factors Affecting Pathologic Complete Response Following Neoadjuvant Chemotherapy in Breast Cancer: Development and Validation of a Predictive Nomogram Radiology. 2021 May;299(2):290-300.

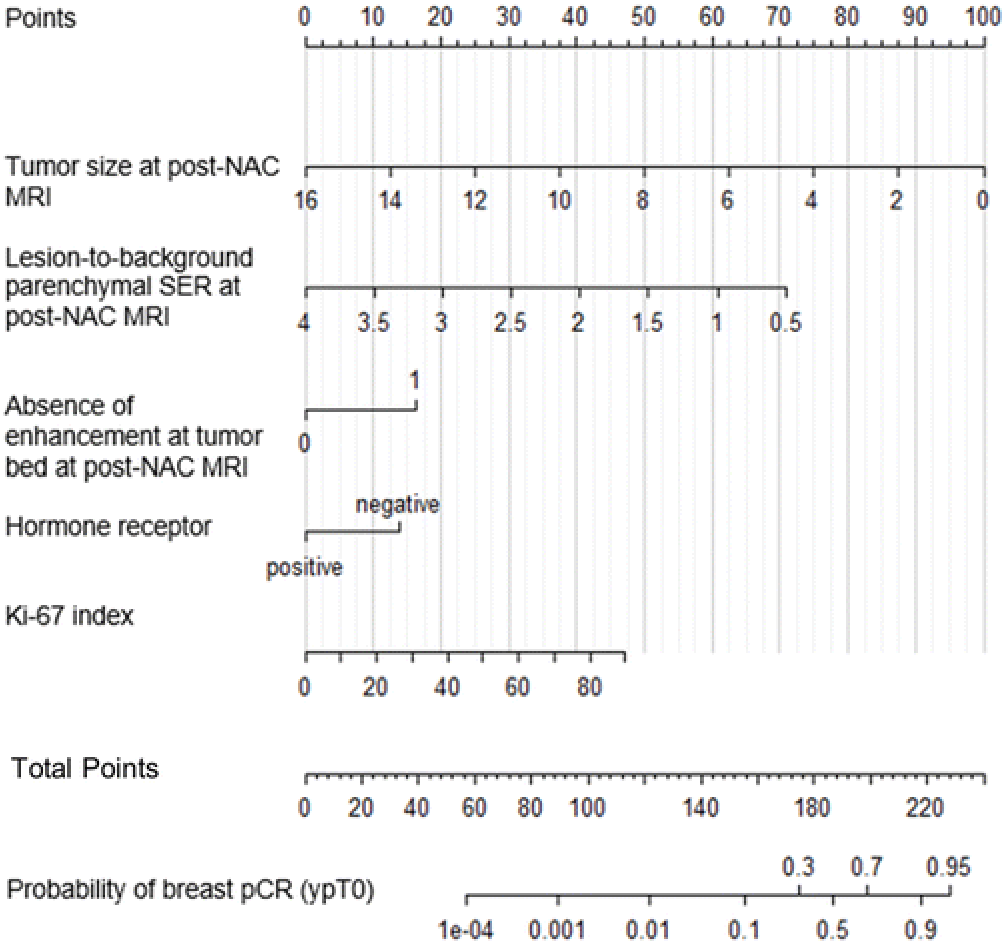

[12]Hwang HW, et al. A nomogram to predict pathologic complete response (pCR) and the value of tumor-infiltrating lymphocytes (TILs) for prediction of response to neoadjuvant chemotherapy (NAC) in breast cancer patients. Breast Cancer Res Treat. 2019 Jan;173(2):255-266.

Fig. 2 **a** Nomogram to predict the probability of pathologic complete response (pCR). *TILs* tumor-infiltrating lymphocytes, *NAC* neoadjuvant chemotherapy, *cN* clinical N stage, *ER* estrogen receptor. **b** A patient of triple-negative breast cancer with cN0 stage and 40% of pre-NAC TILs would have a total of 112 points (44 for 40% pre-NAC TILs, 30 for cN0, and 38 for ER negativity). The predictive value of pCR after NAC for this patient is 57%

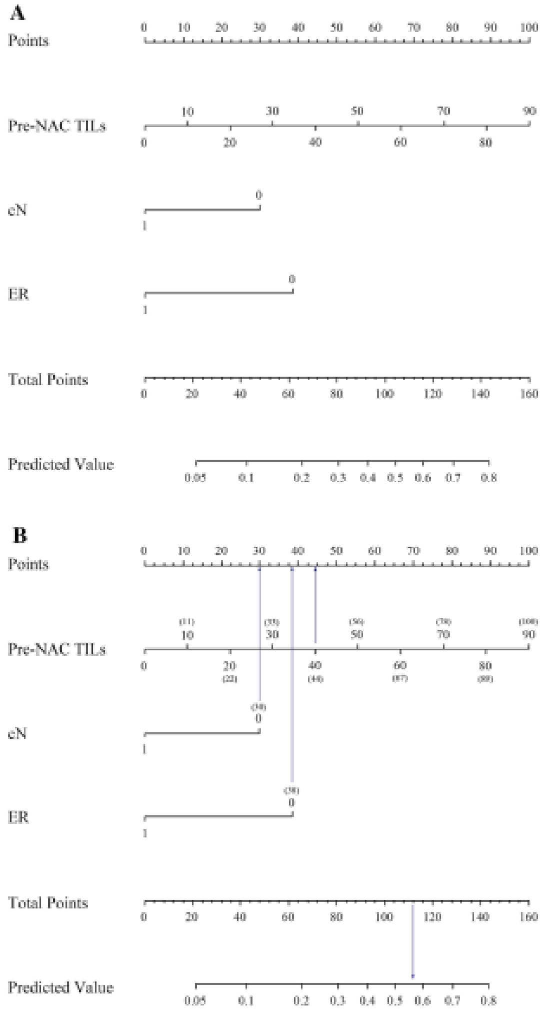

[8]Li Y, et al. A nomogram based on clinicopathological features and serological indicators predicting breast pathologic complete response of neoadjuvant chemotherapy in breast cancer. Sci Rep. 2021 May 31;11(1):11348. doi: 10.1038/s41598-021-91049-x. PMID: 34059778; PMCID: PMC8167133.

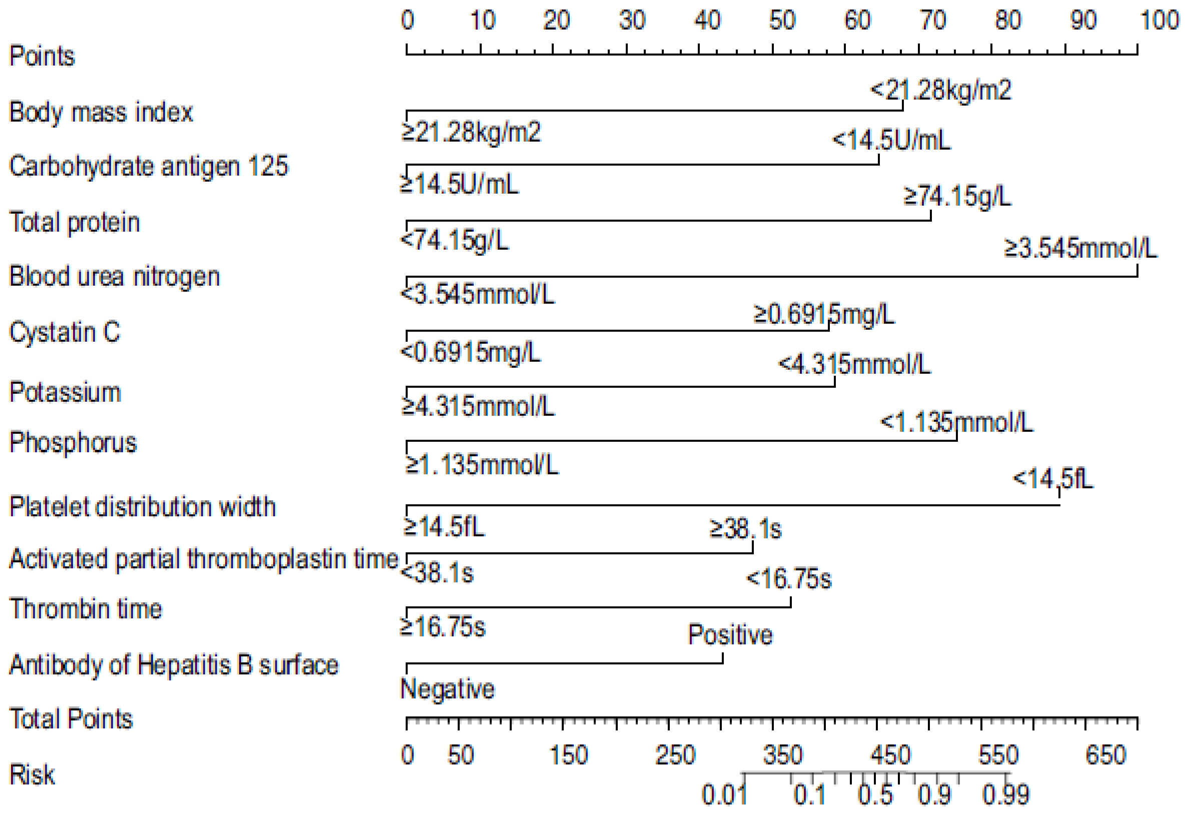

Figure 2. Nomogram to predict breast pathologic complete response rate of breast cancer patients after neoadjuvant chemotherapy based on clinicopathological characteristics and serological indices.

[13]Hou N, Xiao J, Wang Z, Wu Y, Hou G, Guo L, Zhang J, Ling R. Development and Validation of a Nomogram for Individually Predicting Pathologic Complete Remission After Preoperative Chemotherapy in Chinese Breast Cancer: A Population-Based Study. Clin Breast Cancer. 2020 Dec;20(6):e682-e694. doi: 10.1016/j.clbc.2020.06.010. Epub 2020 Jul 8. PMID: 32713825.

Figure 2 Nomogram to Individually Predict pCR Rate Treated With PC for Breast Cancer. Nomogram Is Used by Totaling Points Identified on Top Scale for Each Independent Covariate; Total Points Projected to Bottom Scale Indicate Probability (%) of pCR

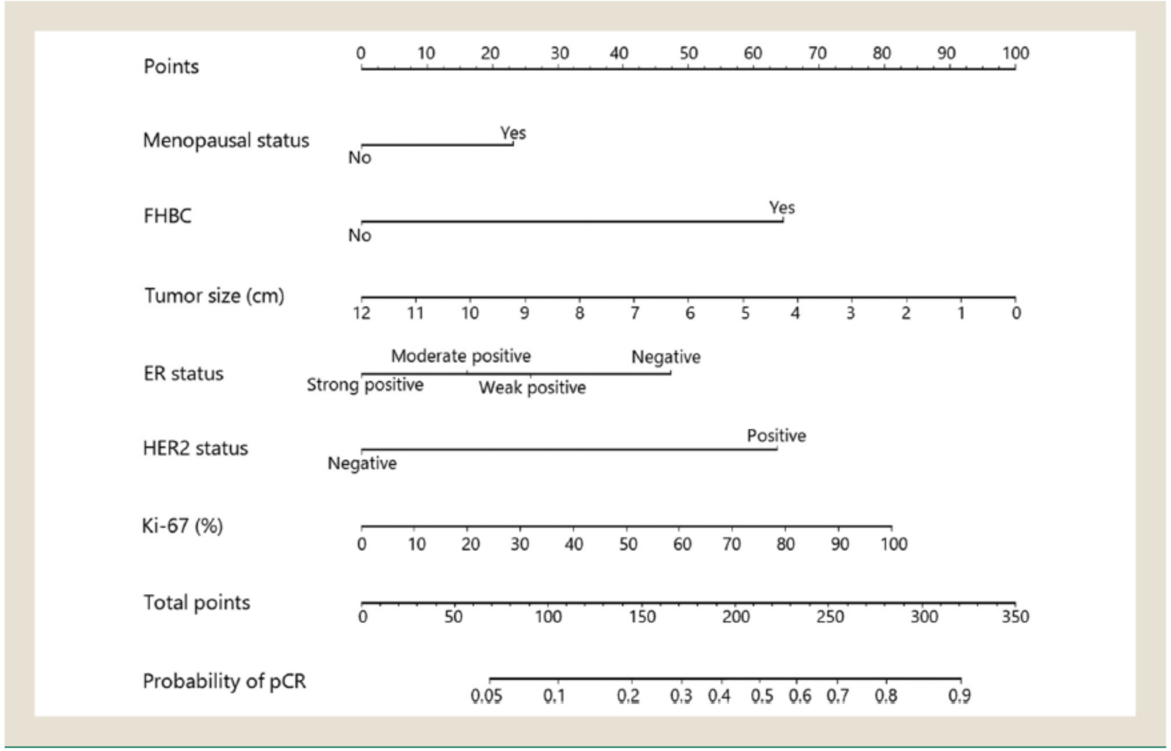

Abbreviations: ER = estrogen receptor; FHBC = family history of breast cancer; HER2 = human epidermal growth factor receptor 2; PC = preoperative chemotherapy; pCR = pathologic complete remission.

[14]Fujii T, Kogawa T, Wu J, Sahin AA, Liu DD, Chavez-MacGregor M, Giordano SH, Raghavendra A, Murthy RK, Tripathy D, Shen Y, Yamal JM, Ueno NT. Nomogram to predict pathologic complete response in HER2-positive breast cancer treated with neoadjuvant systemic therapy. Br J Cancer. 2017 Feb 14;116(4):509-514. doi: 10.1038/bjc.2016.444. Epub 2017 Jan 12. PMID: 28081544; PMCID: PMC5318977.

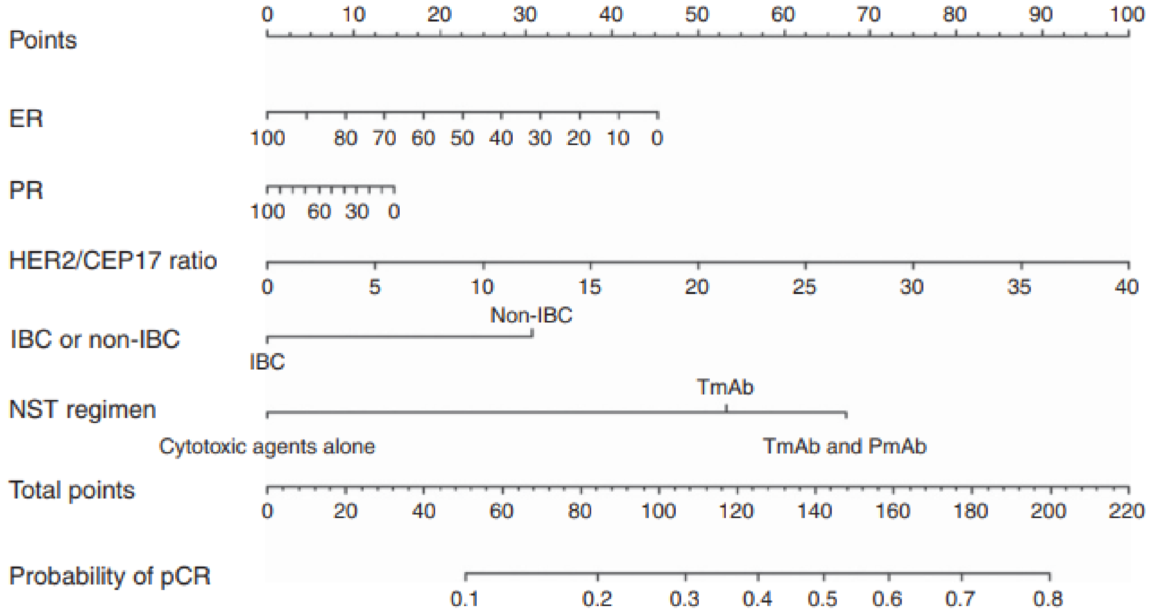

Figure 1. Nomogram to predict the probability of pathologic complete response (pCR). ER = oestrogen receptor; IBC = inflammatory breast cancer; PR = progesterone receptor.

[15]Zhang F, Huang M, Zhou H, Chen K, Jin J, Wu Y, Ying L, Ding X, Su D, Zou D. A Nomogram to Predict the Pathologic Complete Response of Neoadjuvant Chemotherapy in Triple-Negative Breast Cancer Based on Simple Laboratory Indicators. Ann Surg Oncol. 2019 Nov;26(12):3912-3919. doi: 10.1245/s10434-019-07655-7. Epub 2019 Jul 29. PMID: 31359285.

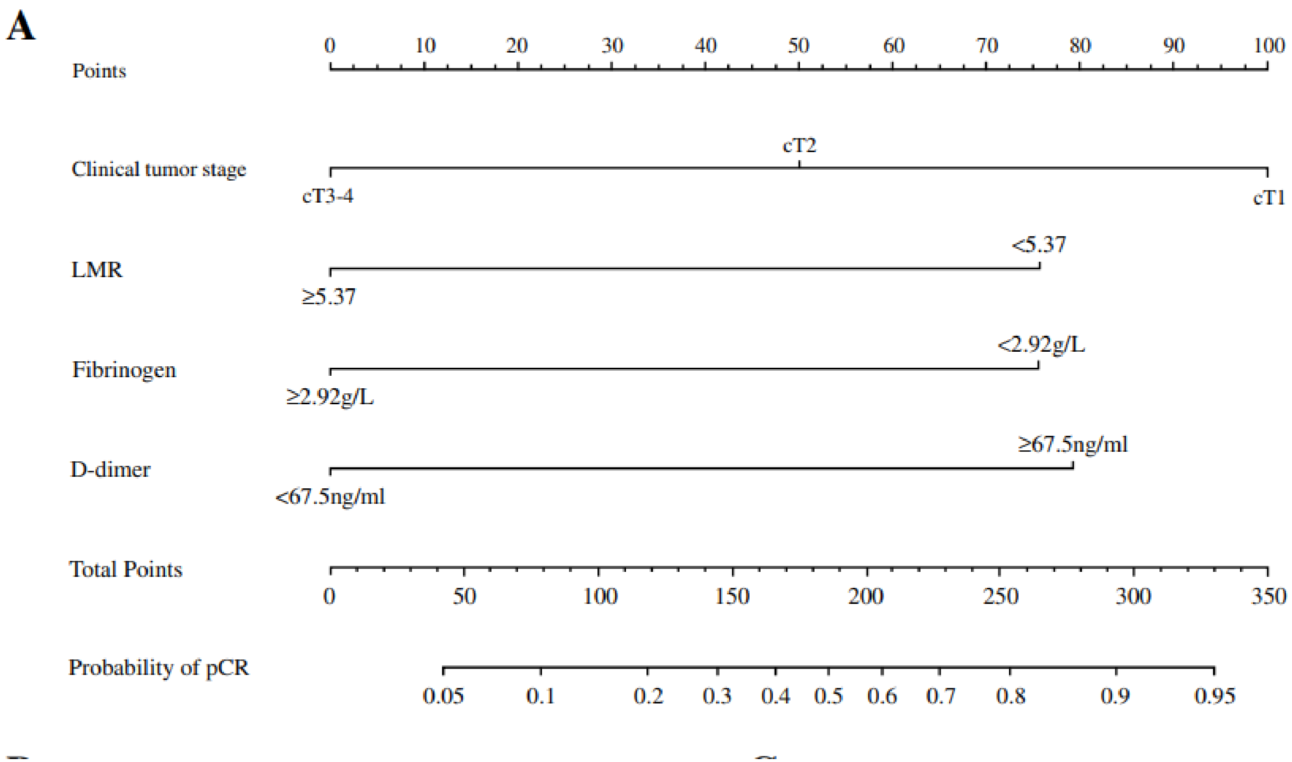

FIG. 1 The nomogram and its calibration and discrimination. **a** The nomogram for predicting the pCR of NAC in TNBC patients. **b** The calibration curve based on internal validation with a bootstrap resampling frequency of 1000. **c** The ROC curve with an AUC of 0.803 to demonstrate the discriminatory ability of the nomogram in predicting the pCR of NAC in TNBC patients. *LMR* lymphocyte to monocyte ratio, *pCR* pathologic complete response, *AUC* area under the curve, *NAC* neoadjuvant chemotherapy, *TNBC* triple-negative breast cancer, *ROC* receiver operating characteristic

[16]Jin X, Jiang YZ, Chen S, Yu KD, Ma D, Sun W, Shao ZM, Di GH. A nomogram for predicting pathological complete response in patients with human epidermal growth factor receptor 2 negative breast cancer. BMC Cancer. 2016 Aug 5;16:606. doi: 10.1186/s12885-016-2652-z. PMID: 27495967; PMCID: PMC4974800.

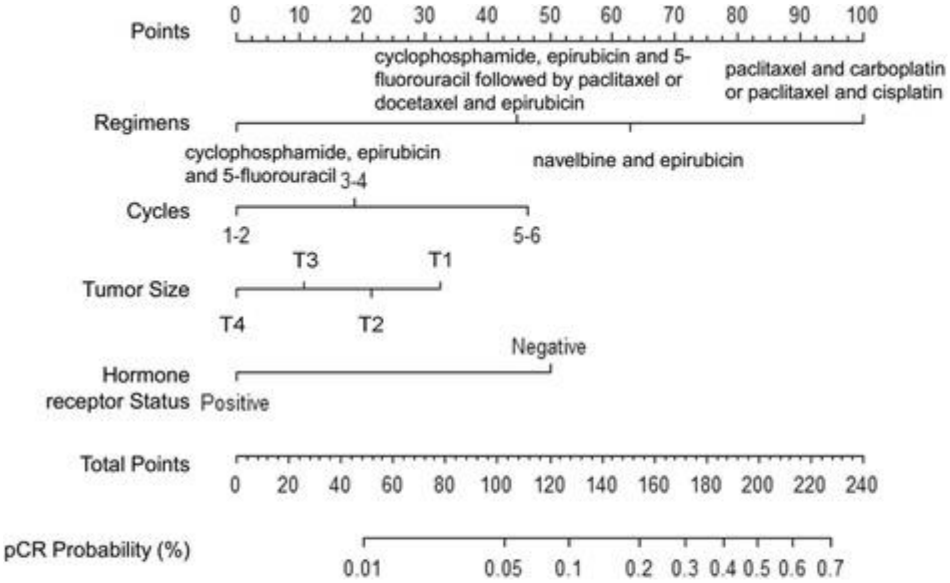

Supplement: Supplementary file 1 [file curroncol-30-00662-s001.zip › curroncol-2637263-supplementary.pdf]
